# Antioxidant Activity of Metal Nanoparticles Coated with Tocopherol-Like Residues—The Importance of Studies in Homo- and Heterogeneous Systems

**DOI:** 10.3390/antiox9010005

**Published:** 2019-12-19

**Authors:** Adrian Konopko, Jaroslaw Kusio, Grzegorz Litwinienko

**Affiliations:** 1 Faculty of Chemistry, University of Warsaw, Pasteura 1, 02-093 Warsaw, Poland; akonopko@chem.uw.edu.pl (A.K.); j.kusio@chem.uw.edu.pl (J.K.); 2 Nencki Institute of Experimental Biology, Polish Academy of Sciences, 3 Pasteur St, 02-093 Warsaw, Poland; 3 Biological and Chemical Research Centre, Faculty of Chemistry, University of Warsaw, ul. Żwirki i Wigury 101, 02-089 Warsaw, Poland

**Keywords:** vitamin E, Trolox, nanoparticles, antioxidant activity, peroxyl radicals, rate constant, micelles, liposomes

## Abstract

Functionalized nanoparticles (NPs) attract great attention in pharmacy, diagnostics, and biomedical areas due to benefits like localization and unique interactions of NPs with biocomponents of living cells. In the present paper, we prepared and characterized two kinds of gold nanoparticles (AuNPs) coated with α-tocopherol-like residues: **1A** were soluble in non-polar solvents and their antioxidant activity was tested during the peroxidation of a model hydrocarbon in a homogeneous system, whereas nanoparticles **1B** were soluble in polar solvents and were applied as antioxidants in micellar and liposomal systems. The effectiveness of **1A** is comparable to 2,2,5,7,8-pentamethylchroman-6-ol (PMHC, an analogue of α-tocopherol). Taking the results of the kinetic measurements, we calculated an average number of 2150 chromanol residues per one NP, suggesting a thick organic coating around the metal core. In heterogeneous systems, the peroxidation of methyl linoleate dispersed in Triton X-100 micelles or DMPC liposomes resulted in the observation that **1B** (545 chromanol residues per one NP) was active enough to effectively inhibit peroxidation in a micellar system, but in a liposomal system, **1B** behaved as a retardant (no clear induction period). The importance of microenvironment in heterogeneous systems on the overall antioxidant activity of nanoparticles is discussed.

## 1. Introduction

Unique physical and chemical properties of metal and metal oxide nanoparticles (NPs) make them desirable products that find industrial applications as catalysts, co-catalysts, and also as intermediates used for the manufacturing of advanced materials. On the other hand, NPs are widely used in biomedical areas like diagnostic assays, gene therapy, drug delivery systems, and molecular imaging [1]. As the majority of nanoparticles are redox active species, special attention is paid to their interaction with organic materials and there is an increasing interest in studies of their (cyto)toxicity [2,3]. One of the most accepted mechanisms of NPs cytotoxicity is their possibility to generate reactive oxygen species which can induce oxidative stress [4,5,6,7,8]. There are several mechanisms underlying the pro-oxidative activity of nanoparticles. For example, a presence of pro-oxidative groups or metal ions make the NPs able to react with molecular oxygen and to generate reactive oxygen species. Other classes of mechanisms include interactions with particular parts of the cells where the core of NP-containing transition metal may be involved in redox-active cycling, causing dysregulation of cell pathways. Also, an accumulation of adsorbed NPs can lead to structural changes of the organelles accompanied by the generation of reactive oxygen species. Pro-oxidant activity frequently depends on size [9,10] and shape [5,11] of nanoparticles that can be rationalized by increasing the number of structural defects in the crystal lattice or by exposition of some specifically ordered atoms at the edge of the nanocrystal, thus increasing the reactivity of a nanoparticle compared to a macroscopic object made of the same material. Pro-oxidative activity can be caused by the adsorbed impurities; therefore, synthesis and purification are crucial for the redox activity of NPs [12].

Several advantageous features of NPs, like effective transport and possibility of targeted localization, make them suitable for biomedical applications. For example, silver nanoparticles (AgNPs) in vivo can get into the cells by diffusion or endocytosis and undergo several processes, including release of compounds from NP surface, aggregation, and surface oxidation, leading to the production of silver cations [13,14]. Moreover, AgNPs inhibit the cell proliferation as a result of interaction with membrane proteins responsible for the activation of signaling pathways [7]. Both AgNPs and silver oxide (as a product of surface oxidation) exhibit great affinity to sulfur functional groups and can bind proteins. In this way, the AgNPs can indirectly modulate the activity of antioxidant enzymes. An emerging area of research in the field of oxidative stress is the use of the nanoparticles either as radical scavengers (nanoparticles possessing redox potential) or as carriers for antioxidant molecules. For example, cerium and yttrium oxides can act as antioxidants themselves [15] and can prevent the increase of Reactive Oxygen Species (ROS) intracellular concentration [16,17] by mimicking the activity of the oxidative enzymes, catalase, or superoxide dismutase [18]’ whereas, other nanomaterials, such as inert gold NPs, have to be functionalized with small molecules of antioxidants to exhibit antiradical activity. Inert metal core can be regarded as a carrier but in some cases, the attachment of a natural antioxidant into the nanoparticle surface can yield a nanomaterial with a new functionality, being a sum or a resultant of the properties of the building blocks. Moreover, the antioxidant attached to nanoparticles might possibly be delivered to the cytoplasm or mitochondria across the cell membrane through pinocytosis and then neutralize ROS [19].

**Figure 1:**
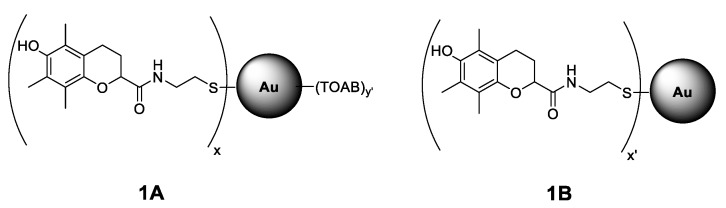
Structure of Au nanoparticles (NPs) coated with chromanol derivative residues stabilized with tetraoctylammonium bromide, TOAB (structure **1A** with TOAB = (C_8_H_17_)_4_N^+^Br^−^) and without additional stabilizer (structure **1B**). The x, x’, and y’ are the symbolic numbers of addends. The presented structures are general and do not correspond to the arrangement of the residues around the nanoparticle.

There are several papers presenting research of synthesis and functionalization of different metallic (mostly gold and silver) nanoparticles having antioxidant activity. Nie et al. [20] reported that gold nanoparticles functionalized with tocopherol analogue exhibit 8 times greater reactivity toward 2,2-diphenyl-1-picrylhydrazyl radicals (**dpph**•) [20]. Gold nanoparticles coated with chitosan exhibited greater activity than ascorbic acid toward hydroxyl radicals formed in an H_2_O_2_/Fe_2_SO_4_ system [21]. Recently, the eco-friendly synthesis of nanoparticles using natural materials was described, with thyme extract used as a reducing and coating agent [22]. Nanoparticles produced by this method showed antibacterial, cytotoxic, and antiradical activity (towards **dpph**•). The same strategy of exploiting the double role of the antioxidant as a reducing and coating agent was applied for silver NPs [23] with glutathione, and the microwave-assisted synthesis resulted in 5–10 nm particles. In order to avoid the oxidation of silver and subsequent release of toxic Ag^+^ ions into the system, other researchers encapsulated AgNPs with a nanothin layer of SiO_2_ (high temperature, gas phase synthesis), and such AgNPs were coated with gallic acid via amide formation between the SiO_2_-NH_2_ moiety and the carboxyl group of gallic acid [24,25]. These nanostructures were 41–42 nm in diameter and due to the presence of gallic acid, they effectively scavenged **dpph**• [26]. Chlorogenic acid entrapped in hybrid materials (SiO_2_ and polyethylene glycol) was a biocompactible nanomaterial able to control ROS formed in the implantation site [27]. An interesting example of enhanced activity of an antioxidant molecule immobilized on NPs was presented by Viglianisi et al. [28], who used graphite layered 30 nm cobalt nanomagnets with attached tocopherol derivatives. The authors studied the ability of the obtained nanostructures to inhibit the autoxidation of styrene at 30 °C and reported that tocopherol derivatives immobilized on the nanomagnets react with alkylperoxyl radicals nine-times faster than free tocopherol analogues (non-attached to CoNPs). Sometimes, the low molecular antioxidant can be immobilized inside the nanomaterial as ascorbic acid loaded into the inner lumen of natural halloysite nanotubes that resulted in increased stability of ascorbate at pH 7.4, and additionally enhanced the number of peroxyl radicals reduced by one molecule of antioxidant [29]. Nonostructural materials like nanotubes offer the opportunity for localization of different antioxidants separated on both sides of the same wall. Such novel synergistic nanoantioxidants were recently described for halloysite externally decorated with tocopherol-like moieties and containing quercetin inside the nanotube [30]. More examples of antioxidants supported by nanoparticles have been described in a recent review [31]. Our previous projects were focused on the antioxidant activity of phenolic antioxidants covalently bonded to fullerene [32,33,34], as we noticed that pristine C_60_ inhibits high-temperature autoxidation of saturated hydrocarbons [35]. We noticed that the C_60_ derivative with covalently bonded hydroxychromanyl moiety (an analogue of α-tocopherol) is an effective antioxidant acting at temperatures above 120 °C during non-isothermal oxidative decomposition of saturated hydrocarbons [34]. In this manuscript, we present the results of our studies focused on the antioxidant activity of tocopherol moieties attached to gold nanoparticles. The inhibiting properties of such functionalized nanoparticles were studied in homogeneous and heterogeneous systems; therefore, two kinds of nanoparticles with different solubility/polarity were prepared and characterized, see Figure 1 presenting the general structures of **1A** (soluble in non-polar solvents) and **1B** (soluble in alcohols).

## 2. Materials and Methods 

### 2.1. Chemicals and Reagents

Chemicals and reagents for synthesis: Cysteamine hydrochloride (98%), Trolox (99%), **b**enzotriazol-1-yloxytris(dimethylamino) phosphonium hexafluorophosphate (BOP) (98%), 4-methylmorpholine (99.5%), 4-dimethylaminopyridine (DMAP) (>99%), tetraoctylammonium bromide (TOAB) (98%), chloroauric acid (99.9%), and solvents were purchased from Sigma‑Aldrich (Steinheim, Germany). Hydroxybenzotriazole (HOBt) (98%) was purchased from Abcer GmbH (Karlsruhe, Germany). Dimethylformamide (DMF) was purified and dried over calcium hydride. Other solvents were of analytical grade and were used as received.

Chemicals and reagents for kinetic measurements: Styrene (≥99%), and cumene (98%), both from Sigma Aldrich (Steinheim, Germany), were percolated on alumina and silica before the experiments in order to remove traces of stabilizer (4-*tert*-butylcatechol). Initiator, α,α’-azobisisobutyronitrile, AIBN (>98% GC, Fluka Chemika, Buchs, Switzerland), was recrystallized from methanol before use. Chlorobenzene (≥99.9% HPLC grade, Sigma Aldrich) was of the highest grade commercially available and used as received. 2,2’-Azobis(2-amidinopropane) dihydrochloride (ABAP; 97%), Triton X-100 (polyethylene glycol p-(1,1,3,3-tetramethylbutyl)-phenyl ether, 98%), and methyl linoleate (LinMe; 99%), 2,2,5,7,8-pentamethylchroman-6-ol (PMHC; ≥99%) were purchased from Sigma-Aldrich (Steinheim, Germany) and were used as received. 1,2-dimyristoyl-*sn*-glycero-3-phosphocholine, DMPC (99%) was from Avanti Polar Lipids, Alabaster, AL, USA), and commercial gold or silver nanoparticles (20 nm diameter AuNPs, AgNPs; BBI Solutions, Crumlin, UK) were stabilized by citrate.

### 2.2. General Information 

^1^H and ^13^C NMR spectra (in CDCl_3_ or D_2_O) were obtained on Bruker Avange 300 MHz spectrometer and edited using MestReNova 10.0 software (Bruker GmbH, Mannheim, Germany). Cary 50 apparatus was applied for UV-Vis measurements (in CHCl_3_, quartz cell, 10 mm optical path). Identification of functional groups by FTIR was performed in solid phase (1 mg of analyte in 300 mg KBr tablets) on a Shimadzu FTIR-8400S spectrometer (Shimadzu Europa GmbH, Duisburg, Germany). Samples for transmission electron microscope (TEM) analysis were prepared on a nickel-coated carbon plate (C_200_Ni_25_, 200 mesh) and measurements were carried out with JEM 1400 JEOL apparatus (JOEL Solutions for Innovation, Peabody, MA, USA) using 120 kV acceleration voltage. Thermogravimetric (TG) measurements were performed using TA Instruments Q50 (calibrated on calcium oxalate monohydrate), with 6 L/h nitrogen flow and 15 °C/min constant heating from 50 to 550 °C. Samples were dried in 40 °C under reduced pressure for 2 h prior to TG measurements. Recorded data were processed with Universal V4.54 TA Instruments software. Dynamic light scattering (DLS) analysis of size distribution of nanoparticles and liposomes was carried out with Malvern Zetasizer Nano–ZS (Malvern, UK), (light source: He-Ne laser 633 nm, detection range 0.3 nm–10 microns) with software provided by the producer.

### 2.3. Synthesis Procedures

**Scheme 1:**
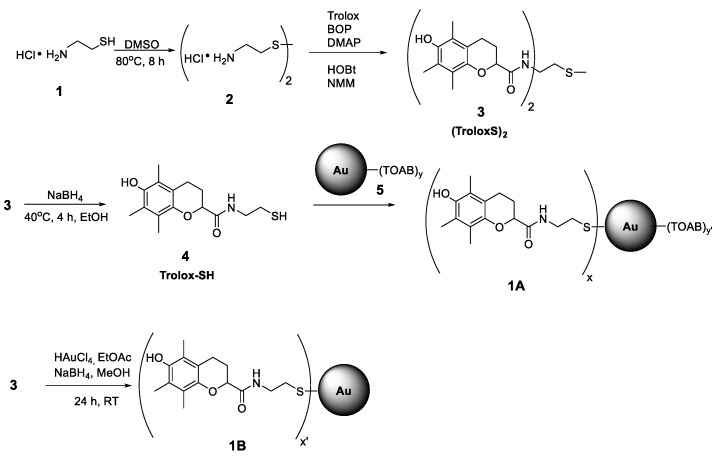
Synthesis pathway for functionalized nanoparticles **1A** and **1B**.

The synthesis pathway from **1** to **1A** (see Scheme 1) was carried out using the procedures described by Nie et al. **[20]** with AuNPs obtained by reduction of chloroauric acid (HAuCl_4_) with sodium borohydride (NaBH_4_) in the presence of tetraoctylammonium bromide (TOAB) (a type of Brust–Schiffrin synthesis) [36].

#### 2.3.1. 2,2′-Diaminodiethyl disulfide dihydrochloride (**2**)

Cysteamine hydrochloride **1** (4.00 g, 35 mmol) was oxidized with 10 mL of DMSO at 80 °C during 8 h [37], and after that time, 5 mL of cold ethanol was added to the solution. The precipitated disulphide was collected and dried under vacuum to give (3.33 g, 85% yield) of white crystals of **2**; mp. 214–218 °C, ^1^H NMR (300 MHz, D_2_O): (ppm) δ3.0 (t, 2H), δ3.4 (t, 2H)), see Figure S1.

#### 2.3.2. *N*,*N*’-(disulfanediylbis(ethane-2,1-diyl))bis(6-hydroxy-2,5,7,8-tetramethylchromane-2-carboxamide) (**3**)

0.55 g of **2** (2 mmol) was dissolved in 10 mL of DMF together with 1.00 g of Trolox (4.0 mmol), 1.95 g (4.4 mmol) of BOP, 0.67 g (4.4 mmol) of HOBt, and DMAP (0.54 g, 4.4 mmol). After 10 min of stirring, 1.45 mL of 4-methylmorpholine (13.2 mmol) was added and the mixture was stirred at room temperature under nitrogen for 18 h. Then, the DMF was evaporated under vacuum in 40 °C, the crude product was dissolved in ethyl acetate and consecutively washed with 0.5 M H_2_SO_4_, Na_2_CO_3_, and brine. The organic layer was then dried with MgSO_4_, concentrated on a rotary evaporator, and purified by column chromatography (silica gel, hexanes‑ethyl acetate 1:1) to yield 0.87 g (70%) pure **3** as white crystal needles. mp. 114–116 °C. ^1^H NMR (300 MHz, CDCl_3_, see Figure S2) δ 1.5 (s, 6H); 1.9 (m, 2H); 2.1 (s, 6H); 2.2 (s, 6H); 2.4 (m, 2H); 2.6 (m, 8H); 3.4 (m, 4H); 6.8 (bs, 2H). ^13^C NMR (75 MHz, CDCl_3_, see Figure S3) δ 11.4; 12.0; 12.3; 20.5; 24.6; 29.6; 37.5; 78.3; 117.9; 119.6; 121.9; 122.0; 144.3; 145.7; 174.7. FTIR (in KBr, see Figure S4): (cm^−1^) 3423 (O–H, stretching), 2926 and 2866 (C–H alkyl, stretching), 1662 (C=O amide, stretching), 1525 (N–H amide, bending), 1452 and 1373 (–C–H alkyl, bending), 1257 (C–O ether, stretching), and 752 (C–S, stretching) and 619 (S–S, stretching). UV-VIS (CCl_3_, see Figure S5) λ_max_ = 285 nm.

#### 2.3.3. 6-hydroxy-*N*-(2-mercaptoethyl)-5,7,8-trimethylchromane-2-carboxamide (**4**)

The standard reduction procedure of compound **3** was used. 100 mg (0.16 mmol) of **3** was dissolved in 5 mL of ethyl alcohol and then NaBH_4_ (12 mg, 0.32 mmol) in 2 mL EtOH was added dropwise with a syringe. The reaction was carried out at 40 °C and the progress of the reduction was monitored by TLC. After 4 h, the reaction was completed and 5 mL of saturated NH_4_Cl aqueous solution was added and the solvents were evaporated under reduced pressure to obtain a volume less than 10 mL. The product was extracted using three portions of ethyl acetate (10 mL each), dried over MgSO_4_, concentrated to 1 mL, and purified by flash chromatography (silica gel, EtOAc). The obtained thiol **4** (82 mg) is not stable, thus, it was immediately dissolved in DCM and used for synthesis of **1A**.

#### 2.3.4. AuNPs Stabilized with TOAB (**5**)

TOAB (383 mg, 0.70 mmol) was added to 5 mL of a stirred aqueous solution of HAuCl_4_ (59 mg, 0.17 mmol), dissolved in 14 mL of toluene, and to this vigorously stirred biphasic system, a solution of 60 mg of NaBH_4_ in water (8 mL) was slowly added at RT. After 2 h, the stirrer was turned off, red colored toluene phase was separated and concentrated under a nitrogen stream, and the obtained nanoparticles were suspended in 5 mL of dichloromethane. The whole amount was used for the synthesis of **1A**.

#### 2.3.5. AuNPs 5 Functionalized with Chromanol Derivative (**1A**)

82 mg of compound **4** dissolved in 5 mL DCM was added to the freshly obtained suspension of **5** in DCM. The mixture was stirred at room temperature for 24 h under N_2_ atmosphere. After this time, the suspension was concentrated and purified by flash chromatography with Sephadex LH-20 (Sigma-Aldrich Co. Steinheim, Germany, elution with DCM/MeOH, 1:1). The product was purified until TLC showed no traces of free **3** or **4**. Nanoparticles **1A** were dried under a vacuum and stored at −20 °C. TEM images of 1A and size histogram are presented in Figures S6 and S7. **1A** are easily suspended in toluene, chlorobenzene, and dichloromethane. In more polar solvents like EtOH, MeOH, and in water, **1A** forms agglomerates. FTIR (in KBr, see Figure S8): (cm^−1^) 3417 (O–H, stretching), 2920 and 2850 (C–H alkyl, stretching), 1647 (C=O amide, stretching), 1506 (N–H amide, bending), 1456 (–C–H alkyl, bending), 1261 (C–O ether, stretching), and 802 (C–S, stretching). UV-VIS in CHCl_2_: λ_max_ = 521 nm and 285 nm, see Figure S9. Thermogravimetric analysis if **1A** is presented in Figure S10. 

#### 2.3.6. AuNPs Functionalized with Chromanol Derivative (**1B**)

This method is the one pot formation of Au nanoparticles and their decoration with Trolox residues, which is the modified methodology proposed for synthesis of AuNPs coated with TEMPO, ie., (2,2,6,6-Tetramethylpiperidin-1-yl)oxyl radical [38]. Solid HAuCl_4_ (145 mg, 0.43 mmol) was added to a solution of **3** (310 mg, 0.50 mmol) in 120 mL ethyl acetate and stirred at room temperature under argon atmosphere. After 10 min, 70 mL of a methanol solution of NaBH_4_ (274 mg) was slowly added. The mixture was stirred during 24 h, partially concentrated, and washed with water. The organic phase was collected, evaporated under reduced pressure, and crude product was purified by flash chromatography (Sephadex LH20, DCM:MeOH 1:1). Nanoparticles of **1B** were dried and stored at −20 °C under argon. Thermogravimetric analysis if **1B** is presented in Figure S11. Size histogram (DSL analysis) is presented in Figure S12. **1B** can easily be suspended in polar solvents like short chain alcohols (EtOH, MeOH, but not in water), and also in H bond-accepting solvents like THF, acetone, and ethyl acetate. In non-polar, non-HB-accepting solvents like toluene, chlorobenzene, dichloromethane, and chloroform, **1B** forms agglomerates with a tendency to precipitation.

### 2.4. Preparation of Micelles

The same methodology as in our previous kinetic studies was used [39]. Glass test-tubes with 10 µL of methyl linoleate (LinMe) and 5.5 mL of 16 mM Triton X-100 were shaken on Vortex for 1 min. Then, 5.5 mL of buffer (pH 4.0 or 7.0) was added and the mixture was shaken again for 1 min. Buffers composition: pH 4.0 acetate (82 mM CH_3_COOH; 18 mM CH_3_COONa) and 7.0 phosphate (25 mM KH_2_PO_4_; 14.5 mM NaOH). The final concentration of lipid and surfactant in the micellar system was 2.74 mM LinMe and 8 mM Triton X-100.

### 2.5. Preparation of Liposomes—Large Unilamellar Vesicles (LUVs)

Large unilamellar vesicles (LUVs) were obtained from multilamellar vesicles (MLVs) by a standard extrusion procedure performed in a small volume extrusion apparatus, as described in our paper on the calorimetric studies of interactions of catecholamines with liposomes [40]. 65.3 mg of 1,2-dimyristoyl-sn-glycero-3-phosphocholine (DMPC) was dissolved in 1.5 mL of chloroform in a round-bottom flask. Subsequently, 4 µL of LinMe was added, solvent was evaporated by a rotary evaporator, and the lipids were vacuum-dried overnight. After that time, the obtained lipid film was suspended buffers in pH 4.0 or 7.0 and the final concentration of lipids was 2.74 mM of LinMe and 20.2 mM DMPC. LUVs were obtained by multiple extrusion (at least 11 times) in an Avanti (Avanti Polar Lipids Inc., Alabaster, AL, USA) mini extruder. The size distribution of liposomes, determined by the DLS method, was 170 ± 45 nm, see Figure S13.

### 2.6. Methodology of Autoxidation Measurements

The antioxidant behavior of nanoparticles was evaluated by monitoring the rate of peroxidation in homogeneous and heterogeneous model systems. For homogeneous systems, the oxygen uptake was followed by pressure transducer apparatus described elsewhere [41,42,43]. Twin 10 mL vessels (a working vessel and reference one) connected to the ultra-sensitive membrane were immersed in a 30 °C water bath. Both vessels contained 4 mL of liquid phase with 4.34 M styrene or 3.56 M cumene and 0.05 M AIBN in chlorobenzene. During the oxidation course, the solution of examined compound was injected into the working vessel to reach the final concentration 2.5–5 μM, while in the reference vessel, 250 μM PMHC was still present in order to completely suppress the peroxidation process (thus, in both vessels, the evolution of N_2_ from AIBN decomposition was compensated). For heterogeneous systems (micelles and liposomes), the uptake of dissolved oxygen during peroxidation was carried out at 37 °C by using a Biological Oxygen Monitor (Yellow Springs Instruments, Yellow Springs, OH, USA) equipped with a Clark-type electrode in the same way as described in one of our previous papers [42,44]. The samples were buffered at pH 4.0 and pH 7.0 with acetate and phosphate buffer, respectively. The chambers with magnetic stirring, containing 5 mL of micelles or 2 mL of liposomes, were saturated with oxygen. The electrode was placed inside the chambers and peroxidation was initiated by injection of methanol solution of 2,2’-azobis(2-methylpropionamidine) dihydrochloride (ABAP, final concentration 10 mM). After 10% of oxygen was consumed, 10 µl of the methanol solution of Trolox, PMHC, **1B**, or aqueous solution of commercial AgNPs/AuNPs stabilized by citrate was added. The amounts of added compounds were adjusted to reach 1 µM concentration of antioxidant (basing on TG results, 0.82 ppm **1B** gives 1 µM concentration of chromanol residues in the sample), or 0.516 µM Au (corresponding to 0.1 ppm Au) for commercial Au nanoparticles stabilized with citrate. The length of inhibition was determined graphically [42]. The methods for calculation of the rate of initiation, rate of constants for inhibition, stoichiometric coefficient, and other details are described in Section 3.2.

## 3. Results and Discussion

### 3.1. Synthesis 

The main goal of the synthetic part of this project was to obtain Au nanoparticles coated with tocopherol-like (chromanol) residues in order to study their antioxidant activity, considered as the ability to trap peroxyl radicals as the main mediators of peroxidation of lipids and hydrocarbons. The two different systems chosen for studies of antioxidant activity (non-polar solvent and biphasic water/lipid systems) prompted us to prepare two kinds of gold nanoparticles coated with chromanol residues, see structures **1A** and **1B** in Figure 1. They differ with polarity and solubility as an effect of the presence or absence of tetraoctylammonium bromide (TOAB), see Section 2.3. Interestingly, both kinds of nanoparticles, **1A** and **1B,** have similar size. The average diameter of the metallic core of nanoparticles **1A** is 4.41 ± 0.65 nm (TEM analysis), with the largest part of population within the range 3.98–4.31 nm (based on the histogram for 300 NPs). TG analysis showed that **1A** contains 7% of solvent residue, ~70% of organic coating, and ~23% of Au residue. The average diameter of **1B** is ≈3.6 ± 0.39 nm (DLS measurements) and ≈3.15 ± 0.40 nm (TEM). TG analysis indicated 36% of organic coating and ≈64% of Au in **1B**.

### 3.2. Methodology of Calculation of Kinetic Parameters

Our goal was to study the antioxidant activity of the obtained nanoparticles not only in homogeneous solutions but also in model heterogeneous systems, because the lipids dispersed as micelles, liposomes, bilayers, and other model membranes are more relevant to biological systems. The kinetics of autoxidation in many of these heterogeneous systems have been shown to follow the same rate law as in homogeneous solutions [45], and below we describe the same methodology of evaluation of the kinetic parameters for both systems.

Thermal decomposition of azo-initiator (AIBN or ABAP) gives a stable flux of primary radicals:

azo-Initiator→ 2*f* Ini• + N_2_   (0 < *f* < 1) (1)

immediately reacting with molecular oxygen and producing more stable peroxyl radicals, IniOO•, able to attack molecules of hydrocarbon (RH = styrene, cumene, or methyl linoleate):

IniOO• + RH→ IniOOH + R•  ; *R*_i_ (2)

This process triggers a propagation chain of consecutive reactions of the addition of oxygen to the alkyl radical (3) and the abstraction of the hydrogen atom from hydrocarbon (4).

R• + O_2_ → ROO•       ; *k*_2_ (3)

ROO• + RH→ ROOH + R•    ; *k*_p_ (4)

Reaction 3 is very fast, with *k*_2_ ~ 10^9^ M^−1^ s^−1^, whereas *k*_p_ for Reaction 4 is within the range 0.3–60 M^−1^ s^−1^ (depending mainly on C–H bond strength in RH) [41,43]. Therefore, Reaction 4 is a rate determining step for propagation during the spontaneous (non-inhibited) peroxidation. The Reactions 3 and 4 are repeated tens and hundreds of times, and thus, hydrocarbon is converted to hydroperoxides until the kinetic chain is terminated during recombination of two radicals, Reaction 5, with *k*_t_ = 2.8 × 10^4^ M^−1^ s^−1^ for cumene and 2.1 × 10^7^ M^−1^ s^−1^ for styrene [41].

2ROO• → non-radical products   ; *k*_t_ (5)

One of the ways to stop the peroxidation at its early step (low conversion) is to break the kinetic chain by adding a compound that will be able to trap peroxyl radicals. Such compounds are called chain-breaking antioxidants, and, for phenolic antioxidants, their action is illustrated by Reactions 6 and 7.

ROO• + ArOH → ROOH + ArO•   ; *k*_inh_ (6)

ROO• + Ar• → non-radical product,(7)

**Figure 2:**
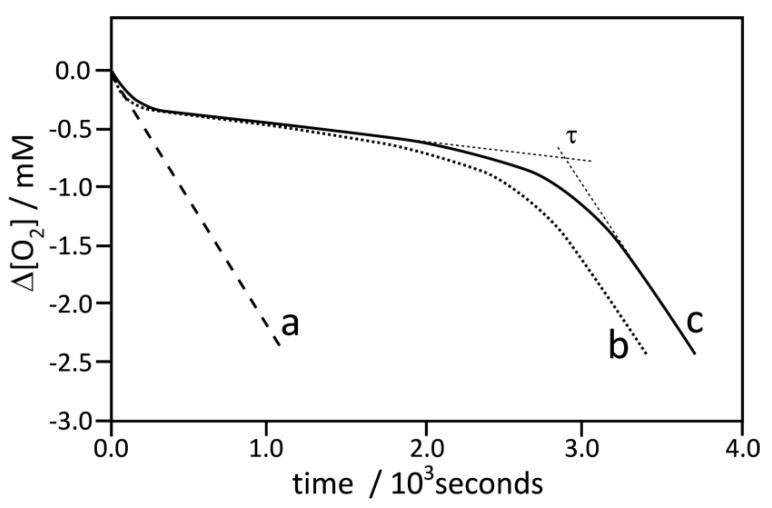
Oxygen uptake profiles during autoxidation of styrene, 4.35 M, in chlorobenzene initiated by 50 mM α,α’-azobisisobutyronitrile (AIBN) in a sample volume 4.0 mL at 30 °C: Curve a, non-inhibited process (the same kinetic trace was also obtained for autoxidation in the presence of nanoparticle **5**), Curve b, inhibited by nanoparticles **1A** at concentration 10.3 μg/mL, and Curve c, inhibited by compound **3** (dimer of chromanol) at concentration 5 μM.

Rate of initiation, *R*_i_, with 50 mM AIBN or with 10 mM ABAP, was determined by the method proposed by Hammond and Boozer [46]. The length of the induction period (τ, see Figure 2) is dependent on the initial concentration of the antioxidant, [ArOH]_0_ and, those parameters, together with the stoichiometric factor *n* (i.e., the number of trapped peroxyl radicals by one molecule of antioxidant) and the rate of initiation *R*_i_, are connected to each other by Equation (8).

τ = *n* [ArOH]_0_/*R*_i_ (8)

After simple transformation of Equation 8 to the form *R*_i_ = *n* × [ArOH]/*τ*_ind_ and using the known concentration of PMHC as an inhibitor (*n* = 2.0), the parameter *R*_i_ can be determined. On the other side, the same equation can be employed for the determination of *n* for a novel antioxidant, when *R*_i_ and τ are known. The inhibition rate constants *k*_inh_ were determined from integral form of the rate equation:

(9) $$\Delta {\left[O_{2}\right]}_{t}=-\frac{k_{p}\;\left[\mathrm{RH}\right]}{k_{inh}}\ln\left(1-\frac{t}{\tau }\right)$$

where: Δ[O_2_]_t_ is molar oxygen consumption [M] recorded at time t within the induction period, *k*_p_ is the rate constant for propagation [M^−1^ s^−1^], [RH] is the concentration of oxidized hydrocarbon, and *τ* is the length of the induction period [s]. The following values of *k*_p_ were taken for calculations: 41 M^−1^s^−1^ for styrene [43,47], 36 M^−1^ s^−1^ for methyl linoleate dispersed in Triton X-100 micelles (assuming the same *k*_p_ as in SDS micelles [48]), and 41 M^−1^ s^−1^ for MeLin in DMPC liposomes [49].

### 3.3. Antioxidant Activity in Homogeneous System

Experiments with Au nanoparticles stabilized with TOAB (Scheme 1, compound **5**) indicated that **5** does not inhibit or retard the autoxidation of styrene. For weak antioxidants, cumene is proposed as a much better substrate that undergoes two orders of magnitude slower autoxidation than styrene (*k*_p_^cumene^ = 0.18–0.32 M^−1^ s^−1^ versus *k*_p_^styrene^ = 41 M^−1^ s^−1^), thus, antioxidants with small *k*_inh_ can noticeably retard the autoxidation due to a much higher ratio of *k*_inh_/*k*_p_ in cumene than in styrene [50]. We tested the kinetic behavior of **5** during the autoxidation of cumene; however, also in this system, AuNPs stabilized with TOAB did not exhibit retardant or antioxidant activity. On the other side, in both hydrocarbons, no pro-oxidant effect of **5** was noticed. Recently, Baschieri and coworkers [12] reported that AuNPs with even traces of onium salts (including TOAB) exhibit clear pro-oxidant behavior. The contradiction between their observations and our results is apparent, but in our system, the autoxidation was initiated with AIBN, while Baschieri et al. used tert-butyl hydroperoxide as an initiator, and they rationalized the observed pro-oxidant effect as a result of catalytic decomposition of tBuOOH induced by onium salts.

Figure 2 presents the typical plot of oxygen uptake during the oxidation of styrene in the presence of nanoparticles **1A** (line b) and in the presence of dimer **3** (line c). In separate experiments with PMHC, the rate of initiation was determined as 5.53 nMs^−1^ (see Figure S14), thus, based on the induction time determined for the process inhibited by 5 µM of **3** (see Table 1), we were able to calculate the stoichiometric coefficient. Taking into account that **3** is a dimer of chromanol, the determined *n* = 3.4 ± 0.2 for **3** (in means 1.7 per OH group) is in reasonable agreement with *n* = 2 for the reference PMHC. The rate constants *k*_inh_ calculated from the kinetic experiment for **3** and **1A** are in good agreement with 6.4 × 10^5^ M^−1^ s^−1^, determined by Amorati et al. [28] for tocopherol in a similar system (styrene/benzonitrile/AIBN). The same authors described enhanced reactivity (ca. nine-fold) of tocopherol residues bound to cobalt nanoparticles (coated with graphite) but we did not observe the enhancement for **1A** compared to **3**. For both species, the *k*_inh_ values in chlorobenzene are close to each other, suggesting the important role of a solvent (chlorobenzeve versus benzonitrile) on the mechanism and reactivity of decorated nanoparticles. Line b in Figure 2 with a clear induction period presents the trace of oxygen uptake for the process inhibited by nanoparticles **1A**. Since AuNPs **5** used alone do not contribute to the kinetics of oxidation (see above), the inhibition effect observed in plot *b* (with nanoparticles **1A**) can be assigned to chromanol residues bonded to the surface of AuNPs. The molar concentration of chromanol residues cannot be calculated directly from TG measurements since NPs are coated not only with chromanol but also with TOAB residues, whose concentration is not precisely known (see Figure 1). We are able to calculate the effective molar concentration of chromanol moieties as 7.8 µM, based on Equation 8, with the assumption that *n* for each single chromanol residue is 1.7 (as a half of *n* determined for **3**, see above) and using τ = 40 min determined for **1A** (Table 1) and with *R*_i_ = 5.53 nMs^−1^.

A simple calculation combining the concentration of **1A** (10.3 μg/mL) with the results of TG analysis (23% of Au in **1A**) gives n_Au_ = 48.0 nanomole of gold in the 4 mL sample. The total amount of chromanol in the same volume is 7.8 µM × 4 mL = 31.2 nmol, and the molar ratio n_chromanol_/n_Au_ is 0.65. Having the average diameter of the metallic core 4.4 nm taken from TEM analysis, we estimated that one nanoparticle contains 3300 atoms of Au [51]; therefore, one Au nanoparticle is covered with 3300 × (n_chromanol_/n_Au_) = 2150 chromanol residues, and it can be concluded that 70% of the organic fraction of **1A** indicated by TG analysis is a sum of ~25% chromanol derivatives and 45% of TOAB. The amount of chromanol residues per surface of **1A** is relatively bigger than the number of 6000 chromanol residues covalently bonded to the graphene-like surface of cobalt nanoparticles (15 nm diameter) described in Reference [28], and also bigger than ca. 400 thiol molecules covering 5 nm AuNPs, as determined by Ravi et al. [51], and bigger than that resulting from the number 4.6 per nm^2^ for maximum packing density of alkanethiols on gold surface [52]. Thus, apart from the first layer, an excess of TOAB and chromanol derivatives have to be present in **1A** as a less organized co-adsorbate. Additional simple calculations give the number of nanoparticles in a 4 mL sample: n_Au_ [mol] × *N*_A_ [atoms/mol] × (3300 atoms/NP)^−1^ = 8.8 × 10^12^ AuNPs.

The calculations presented above demonstrated that it is possible to determine the number of antioxidants bound to metal nanoparticles based on the results of inhibited peroxidation experiments (kinetics and stoichiometry), combined with TG and TEM analysis (information on the metal fraction size of metal core), even for nanoparticles coated with additional, non-antioxidant additives like TOAB.

### 3.4. Antioxidant Activity in Heterogeneous Systems

**Figure 3:**
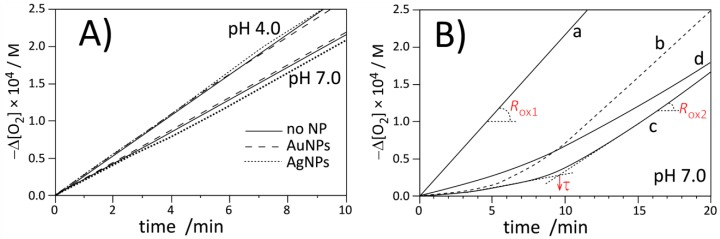
Oxygen uptake for autoxidation of micellar system (2.74 mM LinMe and 8 mM Triton X-100) initiated with ABAP at 37 °C. (**A**) Autoxidation in the absence (no NPs) or presence of commercial AgNPs and AuNPs at pH 4.0 and 7.0. Nanoparticles are stabilized with citrate. (**B**) Autoxidation of micelles at pH 7.0: without inhibitor (curve a), with 1 µM PMHC (curve b, dashed), with 1 µM Trolox (curve c), and with **1B** (equivalent of 1 µM PMHC, curve d).

**Figure 4:**
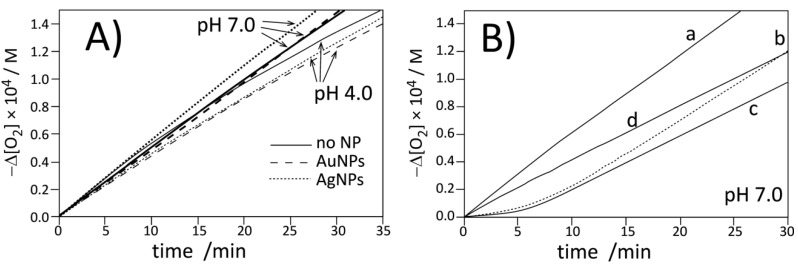
Oxygen uptake for autoxidation of DMPC (20.2 mM) liposomes containing LinMe (2.74 mM) initiated with ABAP at 37 °C. (**A**) Autoxidation in the absence (no NPs) or presence of commercial AgNPs and AuNPs at pH 4.0 and 7.0 (pH indicated by arrows). Nanoparticles are stabilized with citrate. (**B**) Autoxidation of LinMe/liposomes at pH 7.0: without inhibitor (curve a), with 1 µM PMHC (curve b, dashed), with 1 µM Trolox (curve c), and with **1B** (equivalent of 1 µM PMHC, curve d).

Figure 3 and Figure 4 present the plots of the oxygen uptake recorded during peroxidation on methyl linoleate in heterogeneous systems initiated with 10 mM of water-soluble azo-initiator ABAP at pH 4.0 and 7.0 at a temperature of 37 °C. In both systems, micellar and liposomal, we determined the rate of initiation (with PMHC antioxidant, see Figure S15, Figure S16 and Table S1, Table S2) and confirmed [53,54] that *R*_i_ does not vary substantially with increasing pH: *R*_i_ values at pH 4 and 7 in micelles are 4.6 ± 0.2 and 4.4 ± 0.2 nMs^−1^ respectively, and for the same pH values in liposomes, *R*_i_ values are 3.1 ± 0.2 and 3.8 ± 0.4 nMs^−1^. We also monitored the rates of peroxidation in the presence of pristine metal nanoparticles (stabilized with citrate)—as can be seen in Figure 3A and Figure 4A, the kinetic traces are substantially the same (within the experimental error), proving the kinetic neutrality of Ag and Au metal nanoparticles during peroxidation initiated with ABAP.

The kinetic profile of peroxidation of Triton X-100 micelles inhibited by PMHC and Trolox are shown in Figure 3B (lines b and c). The kinetic parameters listed in Table 2 indicate that at the same concentration, PMHC more efficiently suppresses the oxidation during the inhibition period. The rate constant of inhibition, *k*_inh_, for PMHC is in good agreement with the results obtained for PMHC in the same system (2 × 10^4^ M^−1^ s^−1^) [39]. The observed values for PMHC are significantly smaller than *k*_inh_ = 6.9 × 10^5^ M^−1^ s^−1^ determined for **5** in the homogeneous solution (Table 1), and such decreasing reactivity is a result of the kinetic solvent effect (KSE) [54], i.e., because of the formation of an H bond complex between antioxidant (HB donor) and solvent molecules (HB-acceptor), only a fraction of free, non-H bonded antioxidants can react with radicals. KSE was originally discovered and described for H-bond-accepting solvents in homogeneous systems but can be extended for dispersed systems like micelles, emulsions, and liposomes. In our experiments, some fractions of antioxidants were deactivated because their phenolic hydroxyl groups are H-bonded to ether oxygen (in Triton X-100) or to polar head groups of phospholipid (DMPC).

We also observed that Trolox produces a 20% longer induction period compared to PMHC, and, consequently, the stoichiometric factor calculated from the converted Equation 8 for Trolox in micelles is 2.5, in line with the parameters 1.90–2.54 determined by Barclay for Trolox in phospholipid bilayers [55]. On the other side, PMHC more efficiently suppresses peroxidation during the inhibition period (*R*_inh_ is 20%–25% smaller than for the process inhibited by Trolox). The apparent differences in stoichiometry and rates of the process inhibited by those two antioxidants in Triton X-100 micelles can be explained as being due to localization: PMHC is inside the lipid phase while Trolox, as a negatively charged species (carboxylate anion) at pH 7, acts at the lipid/water interface. Some small fractions of Trolox can exist as dianions (with deprotonated phenolic OH moiety) and can scavenge the water-soluble, positively charged peroxyl radicals formed after decomposition of ABAP before they reach the lipid phase [55] and, perhaps, the smaller number of initiating radicals will lead to apparently smaller *R*_i_ (Reaction 2) and, consequently, via Equation 8, to a longer induction period (and bigger stoichiometric factor) for Trolox compared to the same concentration of PMHC. In contrast to the micellar system, in liposomes, Trolox exhibits similar activity to PMHC, see the parameters τ, *R*_inh_, and *k*_inh_ collected in Table 2. Such a substantial change of activity of Trolox in micellar versus liposomal systems at the same pH can be reasoned by coulombic interactions of Trolox anion with positive charges of quaternary ammonium in zwitterionic DMPC membranes. At the moment, we cannot provide a more convincing explanation for such different behavior of Trolox in DMPC and Triton X-100 at the same pH.

Because of solubility reasons, we could not use nanoparticles **1A** for studies of peroxidation of lipids in heterogeneous systems; therefore, nanoparticles **1B** coated with chromanol derivatives (without TOAB) were applied. 10 µL of ethanolic solution of **1B** was injected into a 5 mL sample of micelles or a 2 mL sample of liposomes. Such a small amount of ethanol was not significant for the stability of the heterogeneous system. Measurements carried out with the DLS technique indicated that liposomes containing **1B** were stable within several days and no agglomerates of nanoparticles were formed. Addition of **1B** to liposomes at pH 7.0 was not sufficient to suppress the peroxidation to produce the clear induction period, thus, the experiments with **1B** were not extended onto pH 4.0 and further discussion is limited to the results for pH 7.0 as more biologically relevant. Concentration of **1B** was adjusted to obtain the equivalent of 1 µM Trolox/PMHC based on TG results, indicating 36% of organic coating and with assumption that Au is coated with chromanol residues only. Taking the average diameter 3.15 nm for **1B** (TEM analysis) that corresponds to 1440 atoms [51], it is possible to calculate the average number of 545 chromanol residues per one Au nanoparticle. This number also exceeds 150 residues as calculated for 4.6 molecules of thiol per nm^2^ of gold surface. On the basis of the induction period, using Equation 8 and with the assumption that *n* = 2.5 (determined for Trolox), the concentration of the effectively acting antioxidant is: 558 s × 4.4 nMs^−1^/2.5 = 0.98 µM, that is in perfect agreement (!) with the values calculated separately from thermogravimetric analysis (the same calculations with *n* = 2 instead of 2.5 give 1.23 µM, also with very good agreement to the calculations based on TG analysis). With the number of chromanol derivatives confirmed with two methods, it is obvious that in **1B**, the antioxidants also cover the nanoparticle in the form of multilayer.

The *R*_ox_*/R*_inh_ ratios listed in Table 2 show that the oxidation of methyl linoleate in micelles in the presence of **1B** is four times slower than the non-inhibited process but is also twice as fast as the oxidation inhibited by Trolox. With almost the same induction time for both kinds of inhibitors, it gave a two-fold smaller rate constant for the Reaction of **1B** with peroxyl radicals, compared to *k*_inh_ for Trolox. Since chromanol residues in **1B** are not charged and these nanoparticles are not soluble in water, the observed decrease of *k*_inh_ cannot be attributed to localization of **1B** in water-continuous phase instead of lipid-dispersed phase or water/lipid interface. However, the observed decrease of reactivity (*k*_inh_, *R*_inh_) with the same antioxidant performance (the same stoichiometry as for Trolox) can be interpreted as being due to the limited accessibility of the chromanol residues that are self-assembled in multilayered coating around the metal nanoparticle. In the homogeneous system, the effect of limited accessibility was not detected (see Table 1) and nanoparticles **1A** exhibited the same reactivity as dimers of chromanol **3**. However, dispersed lipid/water systems are dramatically different from homogeneous systems and mobility of nanoparticles in micelles and liposomes is significantly decreased compared to continuous phase, like chlorobenzene solution. 

Moreover, the nanoparticles **1B** that are localized at the lipid water/interface are strongly solvated with water and other polar functional groups, thus, an additional barrier exists and alkylperoxyl radicals cannot reach the deeper parts of the organic coating. Another hypothetical explanation of the same overall stoichiometry for **1B** and Trolox, but different rates of reaction with peroxyl radicals, is that chromanoxyl moieties abstract H atoms from the OH of the chromanols localized in a deeper zone of the coating. The studies of the kinetics of the tocopherol-mediated peroxidation demonstrated that tocopheroxyl radical, if not reduced by co-antioxidant or if not combined with another peroxyl radical, is able to abstract the H atom from lipid in the low-density lipoprotein (LDL) nanoparticle [56,57]. In our system, one Au metal core is surrounded with 545 chromanol residues being in proximity to each other, thus, the H atom transfer from chromanol to the chromanoxyl radical facilitates the participation of the inner sphere in the overall antioxidant activity of **1B**:

(~~Toc–OH)_inner_ +(~~Toc–O•)_outer_ ⇆ (~~Toc–O•)_inner_ +(~~Toc–OH)_outer_ (10)

In this way, **1B** acts slower than Trolox but the stoichiometry indicates that all chromanol residues participate in the antioxidant activity. This hypothesis needs to be verified in future research.

The interactions of chromanol residues with water and the polar part of bilayer in liposomes is even stronger than interactions within micelles; therefore, as can be seen in Figure 4 (panel B, curve *d*) compound **1B** is less reactive toward peroxyl radicals in liposomes and a retardation is observed instead of a clear induction period.

## 4. Conclusions

Using multi-step synthesis, two kinds of gold nanoparticles coated with chromanol residues (tocopherol analogues) were prepared and characterized. Nanoparticles **1A** (23% Au, average diameter of metal core 4.41 ± 0.65 nm) were coated with co-adsorbed tetraoctylammonium bromide (TOAB) to make them soluble in non-polar solvents, whereas nanoparticles **1B** (64% Au, average diameter of metal core 3.15 ± 0.40 nm), coated with pure chromanol residues attached to the metal core, were soluble in methanol and ethanol but unstable in water or in non-polar non-H-bonding solvents.

The rate of peroxidation of styrene in the homogeneous system, initiated with α,α’-azobisisobutyronitrile (AIBN) at 30 °C in chlorobenzene as a solvent, demonstrated that **1A** is an effective antioxidant, comparable to 2,2,5,7,8-pentamethylchroman-6-ol (PMHC, analog of tocopherol). Stoichiometry of antioxidant action determined from the kinetic measurements allowed us to calculate the average number of 2150 chromanol residues per one nanoparticle, suggesting that the arrangement of organic molecules around the metal core is thicker and more complex than the monolayer.

Peroxidations in heterogeneous systems with methyl linoleate as oxidizable lipid dispersed in the micelles of Triton X-100 or in DMPC liposomes (1,2-dimyristoyl-sn-glycero-3-phosphocholine) and initiated with water-soluble 2,2’-azobis(2-amidinopropane) dihydrochloride (ABAP), were followed with a Clark-type oxygen electrode. As a reference compound, PMHC and its water-soluble analog (Trolox) were used. In both systems, the commercial Au and Ag nanoparticles stabilized with citrate were kinetically neutral, whereas nanoparticles **1B** were efficient enough to inhibit peroxidation in the micellar system and to behave as a retardant (no clear induction period) in the liposomal system. The stoichiometry calculated for the reaction of **1B** with peroxyl radicals in the micelles indicates 545 chromanol residues per one nanoparticle, in perfect agreement with the thermogravimetric analysis of **1B**.

The different ability of **1B** to suppress the peroxidation in micelles and in liposomes suggests a great importance of microenvironment in heterogeneous systems. At the water/lipid interface, **1B** is strongly solvated with water and other polar functional groups, causing an additional barrier for penetration of alkylperoxyl radicals into the deeper zone of organic coating. The stoichiometric performance of **1B** to scavenge peroxyl radicals in micelles is very close to Trolox, but **1B** traps peroxyl radicals slower than Trolox. We put forward the hypothesis that peroxyl radicals react with the antioxidants from the outer sphere, with subsequent regeneration of chromanol residues by the phenols from the inner sphere of organic coating. The overall rate of the process is smaller, but stoichiometry is still a sum of stoichiometric coefficients of antioxidants forming the nanoparticle.

## Figures and Tables

**Table 1:** Induction periods (τ) and inhibition rate constants (*k*_inh_) determined during autoxidation of styrene in chlorobenzene at 30 °C initiated with AIBN, in the presence of compounds: **3** and nanoparticles **1A**.

Compound	Concentration	τ/min	10^−5^ × *k*_inh_/M^−1^ s^−1^
**3**	5 µM	48 ± 5 *^a^*	6.93 ± 0.31
**1A**	10.3 ppm *^b^*	40 ± 3 *^a^*	6.95 ± 0.36

*^a^*The average value from at least three measurements ± standard deviation. *^b^*That corresponds to 10.3 μg/mL, but the effective molar concentration of chromanol residues was calculated to be 7.8 µM, see the main text.

**Table 2:** The lengths of induction periods, *τ*, the rates of initiation, *R*_i_, the slow-down factor (*R*_ox_*/R*_inh_, the ratio of the rate of non-inhibited process to the rate of inhibited process), and the inhibition rate constants, *k*_inh_, calculated for autoxidation of 2.74 mM MeLin dispersed in Triton X-100 micelles (8 mM) and in liposomes (20.2 mM), inhibited by 1 µM PMHC, 1 µM Trolox, or **1B** (equivalent of 1 µM PMHC, calculated from TG measurements). In both systems, the experiments were performed at 37 °C and pH 7.0. Peroxidation was initiated by 10 mM ABAP. Each measurement was run 4–6 times. Values are expressed as the mean ± standard deviation (SD).

Experimental System	τ/min	*R*_i_*^a^*/nMs^−1^	*R*_inh_/nMs^−1^	*R*_ox_ */R*_inh_ *^b^*	10^−3^ × *k*_inh_/M^−1^ s^−1^
*micelles*					
PMHC	7.6 ± 0.7	4.4 ± 0.4	37 ± 5	9.0	18.8 ± 3.8
Trolox	9.6 ± 0.4	4.4 ± 0.4	47 ± 8	7.4	4.5 ± 0.9
**1B**	9.3 ± 0.6	4.4 ± 0.4	87 ± 5	4.0	2.2 ± 0.4
*liposomes*					
PMHC	8.6 ± 0.6	3.8 ± 0.4	20 ± 6	5.0	13.9 ± 2.7
Trolox	8.7 ± 0.3	3.8 ± 0.4	24 ± 2	4.2	13.4 ± 2.7
**1B**	-	3.8 ± 0.4	67 ± 3 *^c^*	1.5	-

*^a^* For *R*_i_ determination, see Equation 8 in Section 3.2. *^b^*The (*R*_ox_*/R*_inh_) ratio informs how many times the inhibited oxidation is slower than spontaneous (non-inhibited) process. It can be also considered as the ratio of the kinetic chain length (the number of propagating cycles) of the inhibited to non-inhibited process. *^c^* For this system, the inhibition period was not detected (See curve d in Figure 4B) and the rate of the retarded process is listed.

## Supplementary Materials

The following are available online at https://www.mdpi.com/2076-3921/9/1/5/s1: Figure S1: ^1^H NMR spectrum of the disulphide of cysteamine hydrochloride, Figure S2: ^1^H NMR spectrum of (TroloxS)_2_, Figure S3: ^13^C NMR spectrum of (TroloxS)_2_, Figure S4: FT-IR spectrum of (TroloxS)_2_, Figure S5: UV-Vis spectrum of (TroloxS)_2_, Figure S6: TEM images of **1A**, Figure S7: Size histogram of **1A**, Figure S8: FT-IR spectra comparison: **1A** vs. (TroloxS)_2_, Figure S9: UV-Vis spectrum: **1A**, Figure S10: TG Analysis of **1A**, Figure S11: TG Analysis of **1B**, Figure S12: Size histogram of **1B** obtained by using DLS method, Figure S13: Size histogram of DMPC liposome obtained by using DLS method, Figure S14: Oxygen consumption plots during styrene autoxidation process, Figure S15: Oxygen uptakes curves for autoxidation of LinMe in Triton X-100 micelles in presence of 1 µM PMHC at 37 °C at pH 4.0, Figure S16: Oxygen uptakes curves for autoxidation of LinMe in DMPC liposome in presence of 1 µM PMHC. Measurement were carried out at 37 °C at pH 4.0, Table S1: The kinetic parameters determined for peroxidation of MeLin/Triton X-100, Table S2: The kinetic parameters determined for peroxidation of MeLin/DMPC.

## Abbreviations:

ABAP	2:2’-azobis(2-amidinopropane)
AgNPs	silver nanoparticles
AIBN	α,α’-azobisisobutyronitrile
AuNPs	gold nanoparticles
BOP	benzotriazol-1-yloxytris(dimethylamino) phosphonium hexafluorophosphate
DLS	Dynamic Light Scattering
DMAP	4-dimethylaminopyridine
DMPC	1,2-dimyristoyl-*sn*-glycero-3-phosphocholine
**dpph**•	2,2-diphenyl-1-picrylhydrazyl radical
HOBt	hydroxybenzotriazole
LinMe	methyl linoleate
NPs	nanoparticles
PMHC	2,2,5,7,8-pentamethylchroman-6-ol
R•	alkyl radical
ROO•	peroxyl radical
ROS	Reactive Oxygen Species
TEM	transmission electron microscopy
TGA	thermogravimetric analysis
TOAB	tetraoctylammonium bromide
Triton X-100	polyethylene glycol p-(1,1,3,3-tetramethylbutyl)-phenyl ether
Trolox	6-hydroxy-2,5,7,8-tetramethylchroman-2-carboxylic acid
