# Supplementary material for: Antioxidant Activity of Metal Nanoparticles Coated with Tocopherol-Like Residues—The Importance of Studies in Homo- and Heterogeneous Systems
Source: Antioxidants (Basel). 2019 Dec 19;9(1):5. doi: 10.3390/antiox9010005 (PMC7022694; doi:10.3390/antiox9010005)
Supplement: Supplementary file 1 [file antioxidants-09-00005-s001.pdf]

Supplementary Material

Antioxidant activity of metal nanoparticles coated with tocopherol-like residues – the importance of studies in homo- and heterogeneous systems

Adrian Konopko[1,2;a] , Jaroslaw Kusio[1;a] & Grzegorz Litwinienko[1*]

[a] *A. Konopko and J. Kusio contributed equally to this work*

[1] Faculty of Chemistry, University of Warsaw, Pasteura 1, 02-093 Warsaw, Poland

[2] Nencki Institute of Experimental Biology, Polish Academy of Sciences, Pasteur 3, Warsaw, 02-093, Poland

* Correspondence: litwin@chem.uw.edu.pl

TABLE OF CONTENTS

Title		page
**Figure S1**	^{1}H NMR spectrum of the disulphide of cysteamine hydrochloride.	S-2
**Figure S2**	^{1}H NMR spectrum of $(TroloxS)_2$.	S-2
**Figure S3**	^{13}C NMR spectrum of $(TroloxS)_2$.	S-3
**Figure S4**	FT-IR spectrum of $(TroloxS)_2$.	S-3
**Figure S5**	UV-Vis spectrum of $(TroloxS)_2$.	S-4
**Figure S6**	TEM images of **1A**.	S-4
**Figure S7**	Size histogram of **1A**.	S-5
**Figure S8**	FT-IR spectra comparison: 1A vs. $(TroloxS)_2$.	S-5
**Figure S9**	UV-Vis spectrum: **1A**.	S-5
**Figure S10**	TG Analysis of **1A**.	S-6
**Figure S11**	TG Analysis of **1B**.	S-6
**Figure S12**	Oxygen consumption plots during styrene autoxidation process.	S-7
**Figure S13**	Size histogram of DMPC liposome obtained by using DLS method.	S-7
**Figure S14**	Size histogram of 1B obtained by using DLS method.	S-7
**Figure S15**	Oxygen uptakes curves for autoxidation of LinMe in Triton X-100 micelles in presence of 1 μM PMHC at 37°C at pH 4.0.	S-8
**Figure S16**	Oxygen uptakes curves for autoxidation of LinMe in DMPC liposome in presence of 1 μM PMHC. Measurement were carried out at 37°C at pH 4.0.	S-8
**Table S1**	The kinetic parameters determined for peroxidation of MeLin/Triton X-100.	S-8
**Table S2**	The kinetic parameters determined for peroxidation of MeLin/DMPC.	S-10

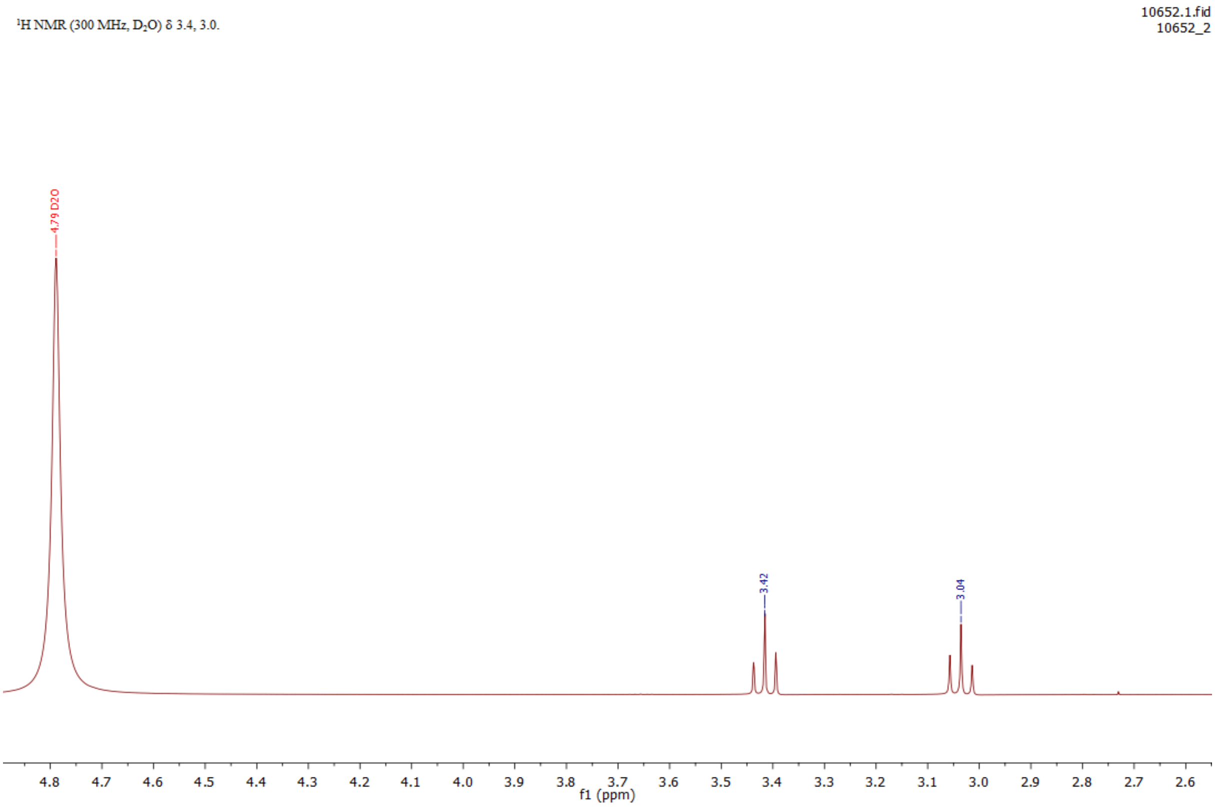

Figure S1. ¹H NMR (300 MHz, D_2O) spectrum of the disulphide of cysteamine hydrochloride - compound **2** in Scheme1 in the main manuscript.

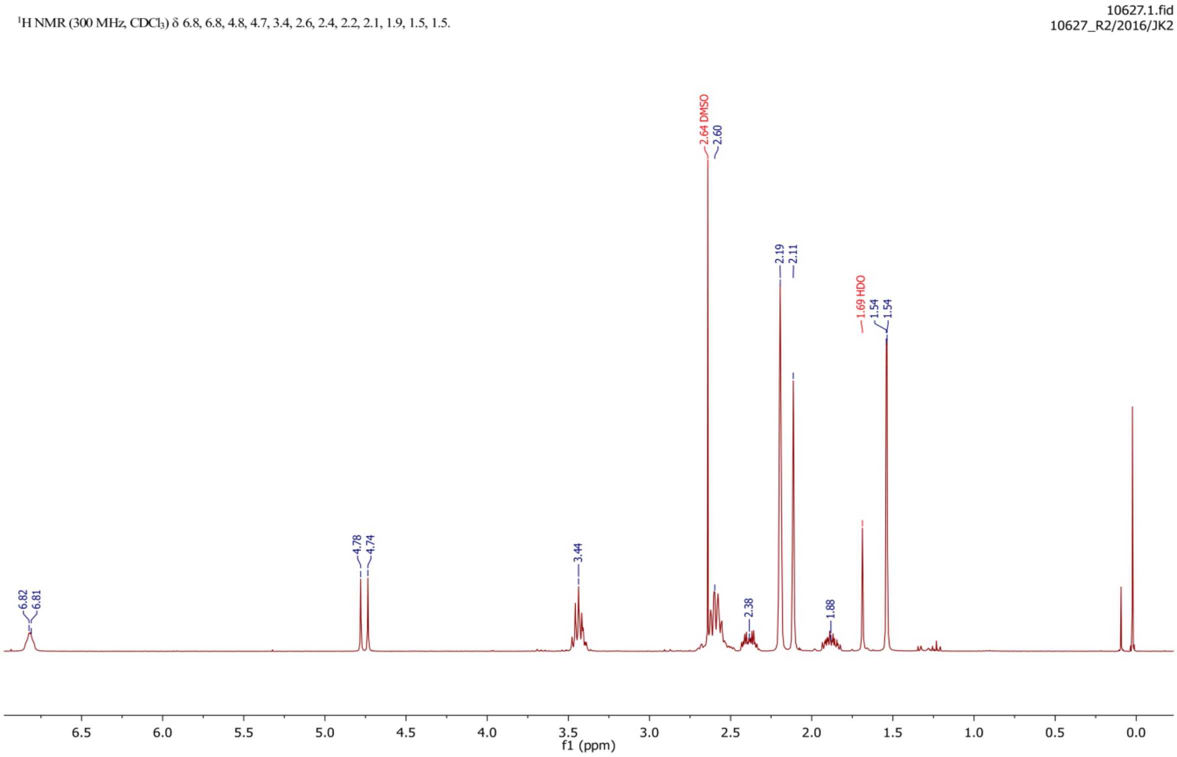

Figure S2. ¹H NMR (300 MHz, $CDCl_3$) spectrum of $(TroloxS)_2$ - compound **3** in Scheme1 in the main manuscript.

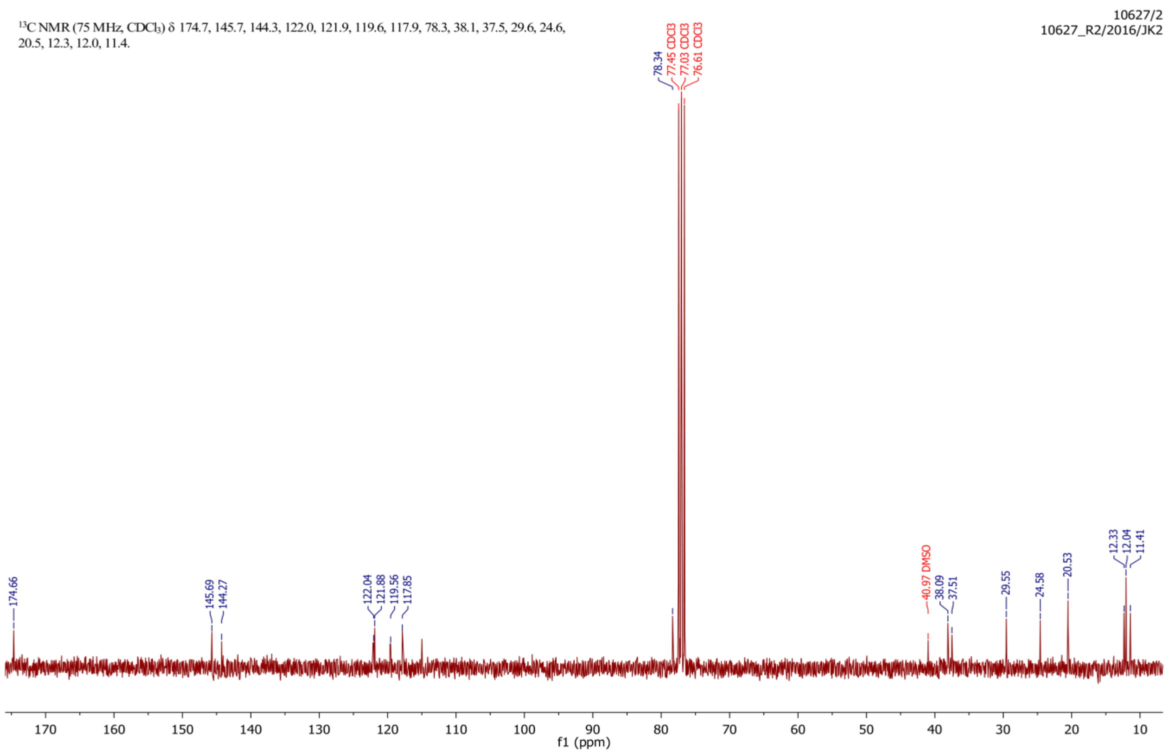

Figure S3. ^{13}C NMR (75 MHZ, $CDCl_3$) spectrum of $(TroloxS)_2$ – compound **3** in Scheme1 in the main manuscript.

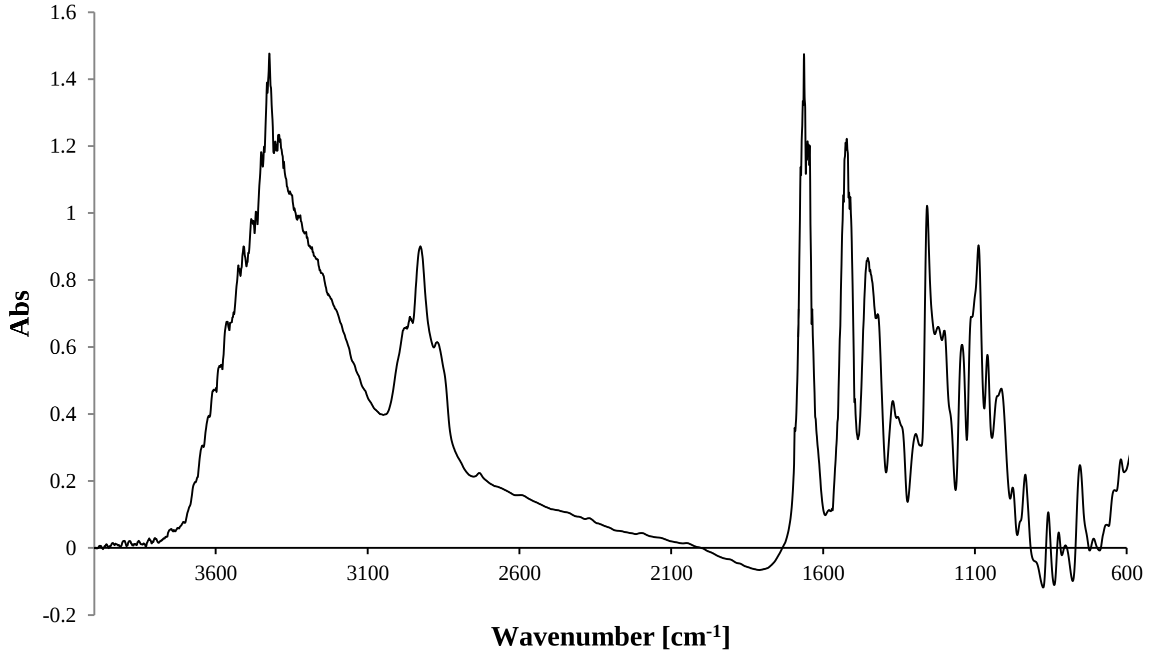

Figure S4. FT-IR spectrum of $(TroloxS)_2$- compound **3** in Scheme1 in the main manuscript.

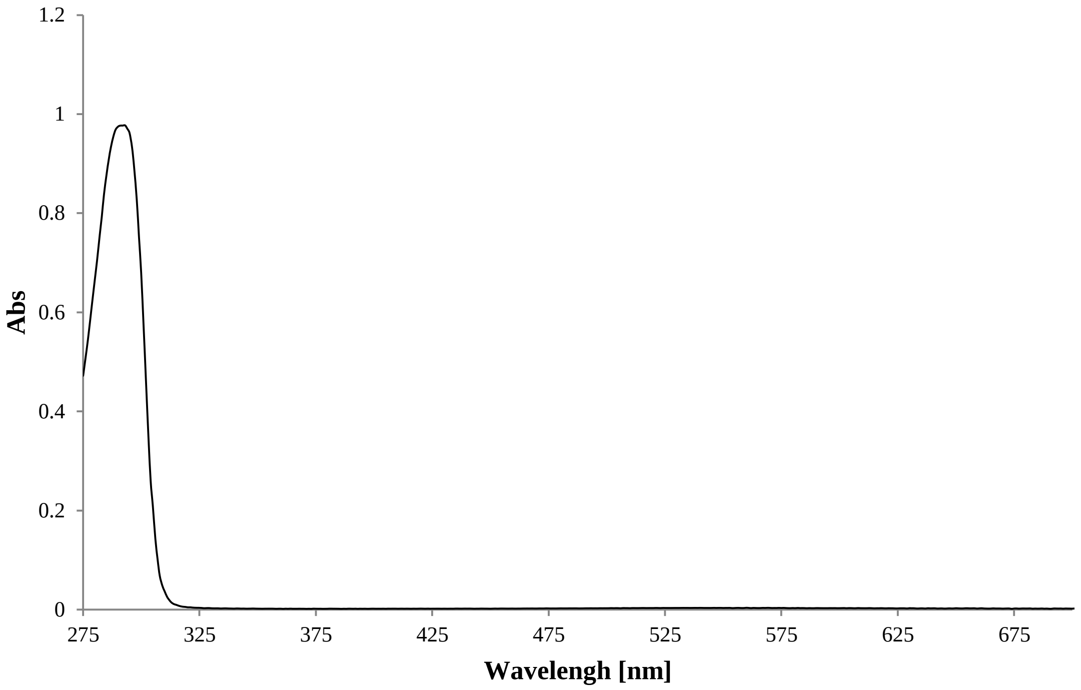

Figure S5. UV-Vis (EtOH) spectrum of (TroloxS)2- compound **3** in Scheme1 in the main manuscript.

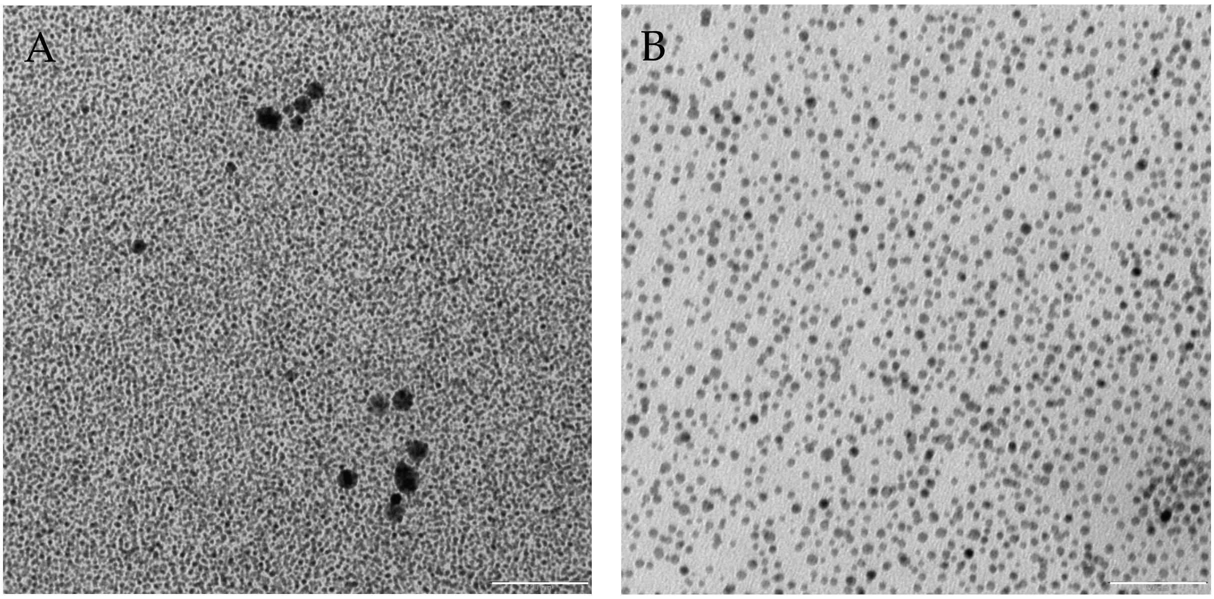

Figure S6. TEM images(A – scale:100 nm, B – 50 nm) of **1A**.

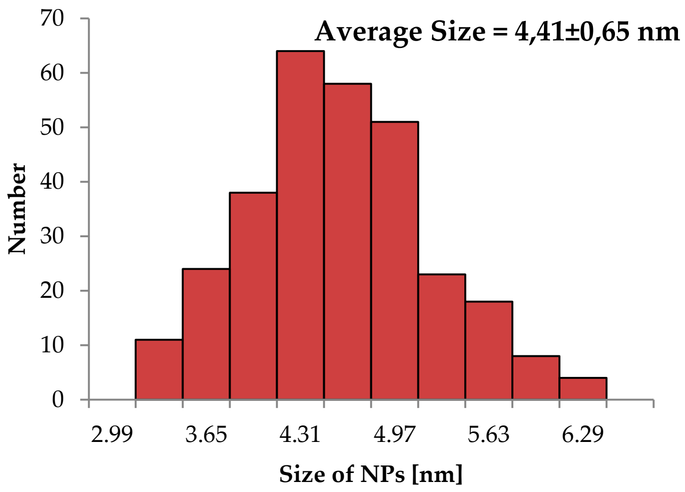

Figure S7 Size histogram of **1A**.

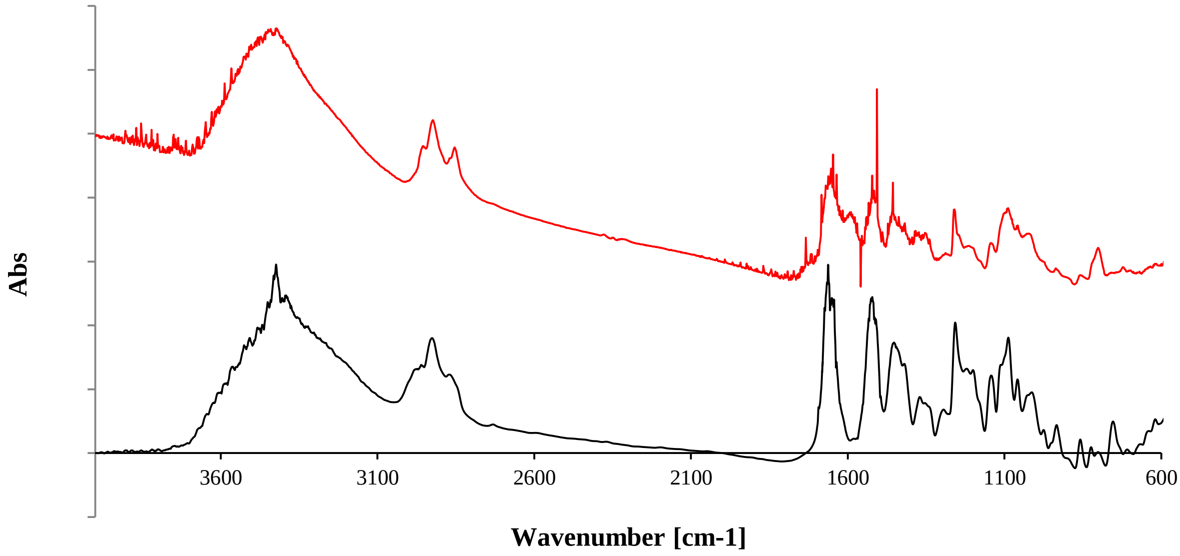

Figure S8. FT-IR spectra comparison: **1A** vs. (TroloxS)$_2$.

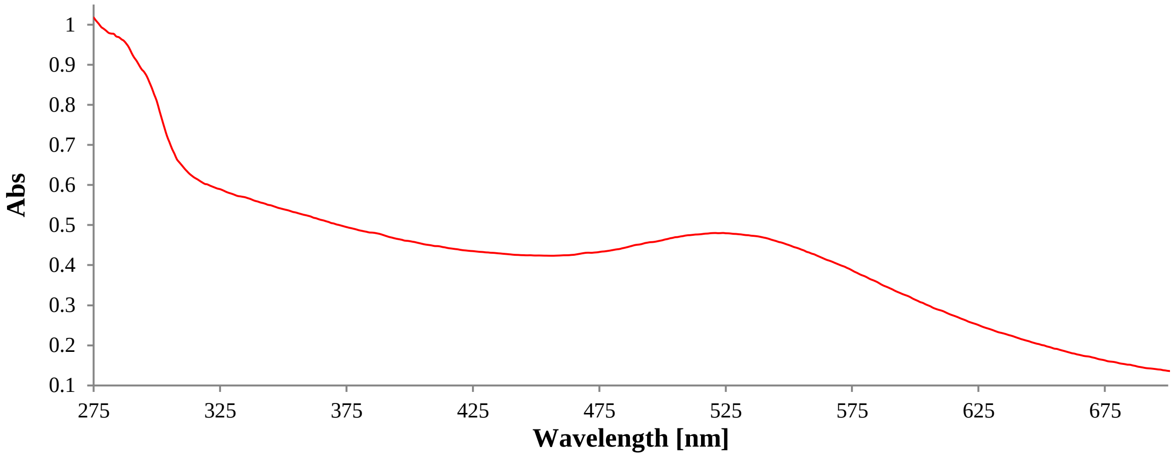

Figure S9. UV-Vis (DCM) spectrum of **1A** (λ_{max}: 521 nm and 285 nm).

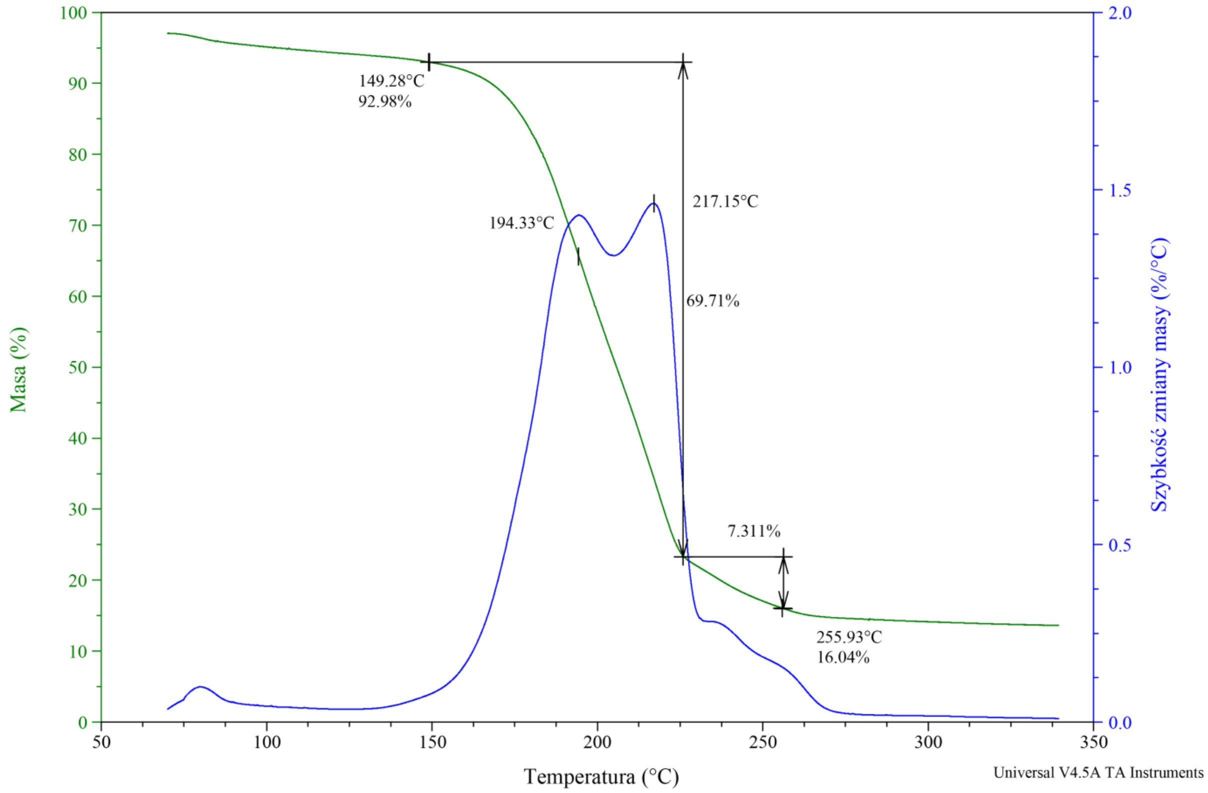

Figure S10. Thermogravimertic plot of **1A**. Starting weight of the sample was 4.19 mg.

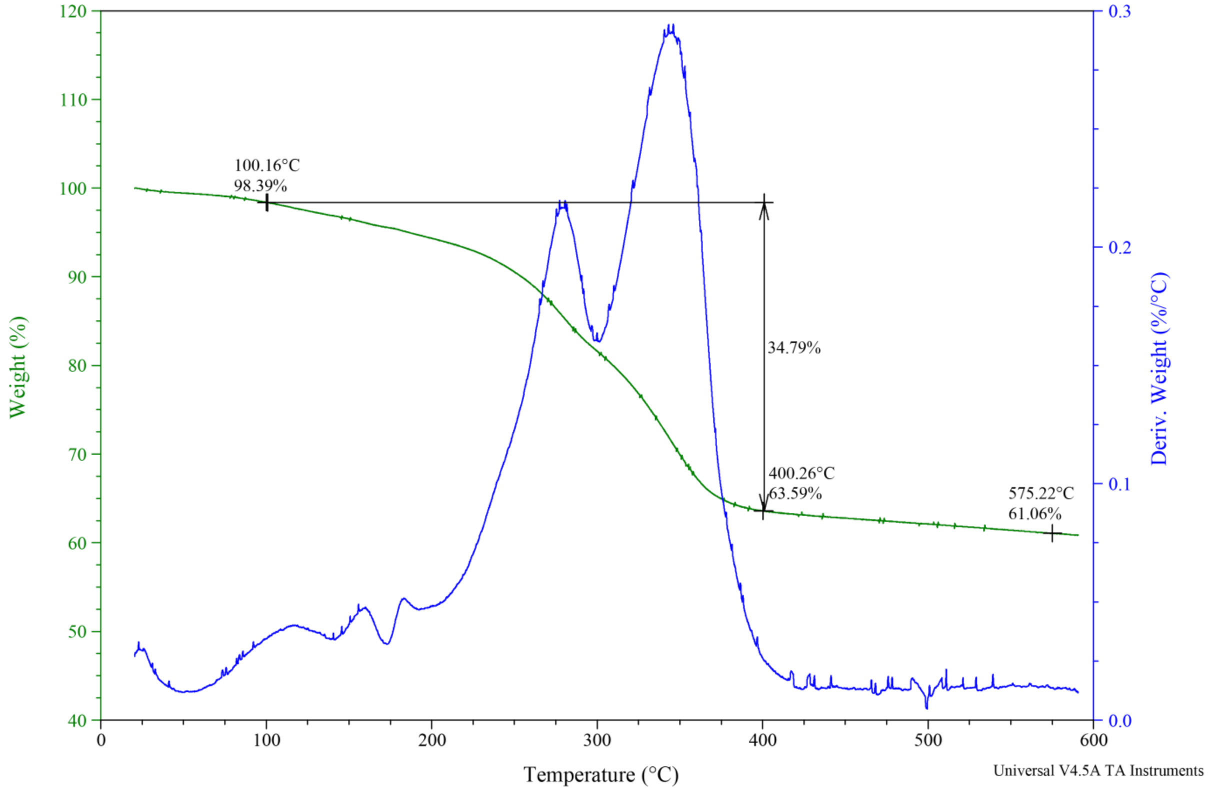

Figure S11. Thermogravimertic plot of of **1B**. Starting weight of the sample was 2.33 mg.

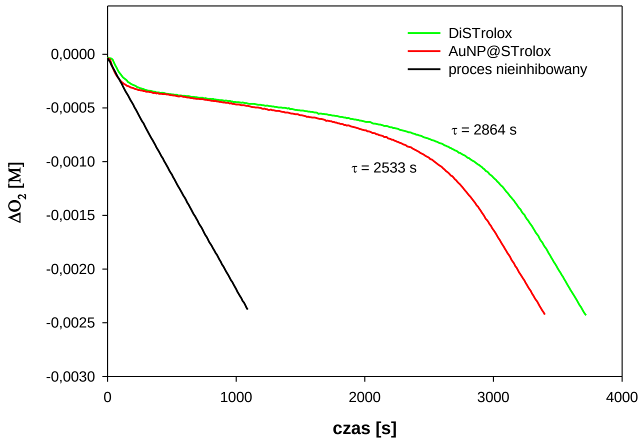

Figure S12. Oxygen consumption plots during styrene autoxidation process.

Sample containing: $(TocS)_2$, 1A, black line – uninhibited process ,τ – induction period [s].

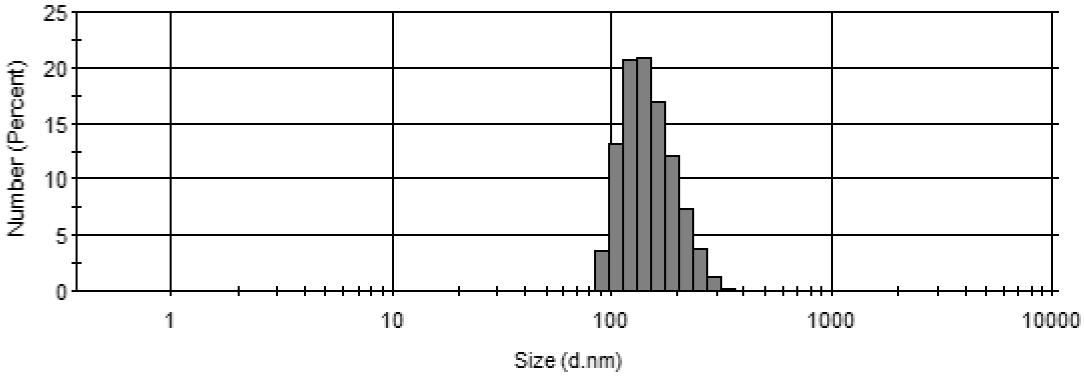

Figure S13. Size histogram of DMPC liposome obtained by DLS method.

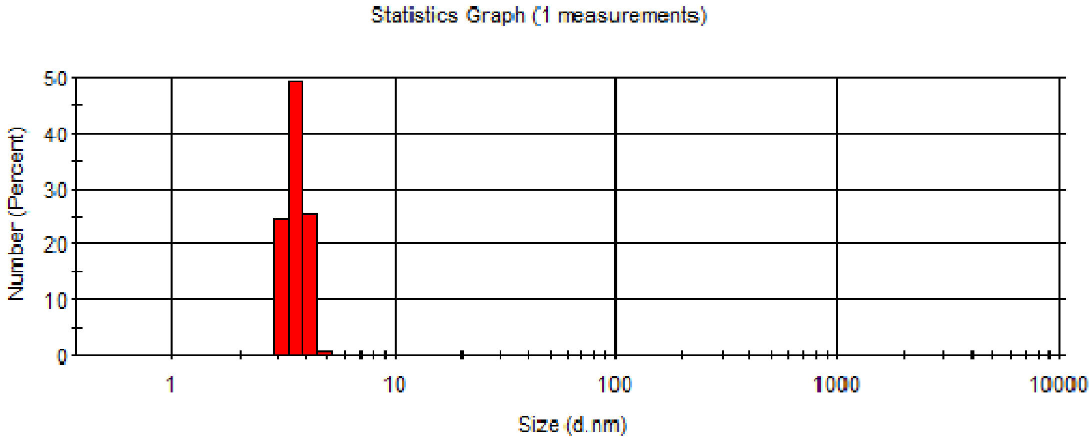

Figure S14. Size histogram of **1B** obtained by DLS.

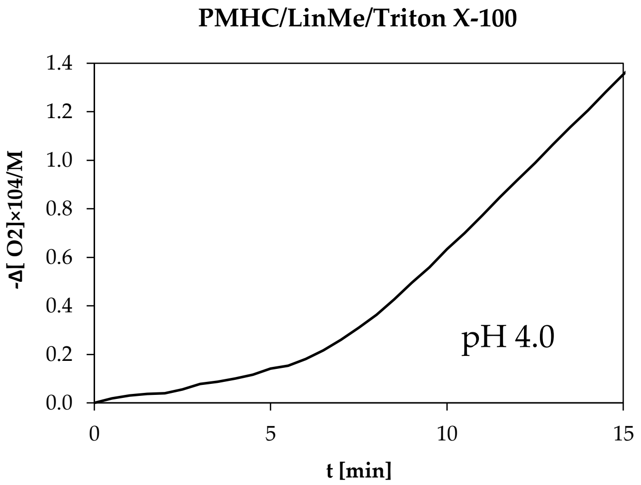

Figure S15. Typical plot of the oxygen uptake for ABAP-initiated autoxidation of LinMe (2.74 mM) in Triton X-100 (8 mM) micelles in presence of PMHC (1 μM). Measurement were carried out at 37°C at pH 4.0.

Table S1. The lengths of induction periods, τ_{ind}, the rates of initiation, R_i, kinetic chain length, v_{ox1}, and the inhibition rate constants, k_{inh}, determined for peroxidation of MeLin/Triton X-100 micelles inhibited by 1 μM PMHC. Experiments were performed in 8 mM Triton X-100 micelles with 2.74 mM MeLin at 37°C at pH 4.0. Peroxidation was initiated by 10 mM ABAP. All experiments were run at 3-6 times. Values were expressed as the mean ± standard deviation (SD).

pH	τ /min	R_i /nMs^{-1}	R_{inh} /nM^{-1}	k_{inh} × 10^{-3} /M^{-1}s^{-1}	R_{ox} × 10^7 /M^{-1}	R_{ox1} × 10^7 /M^{-1}	v_{ox}[a]	v_{inh}[a]	v_{ox1}[a]
4.0	7.2±0.1	4.6	34.5±4.6	10.9±2.2	5.5±0.4	2.4±0.3	120	8	52

[a] The kinetic chain length v is the number of peroxidation cycles triggered by one initiating radical. Here, for non-inhibited peroxidation, $v_{ox1}=R_{ox1}/R_i$

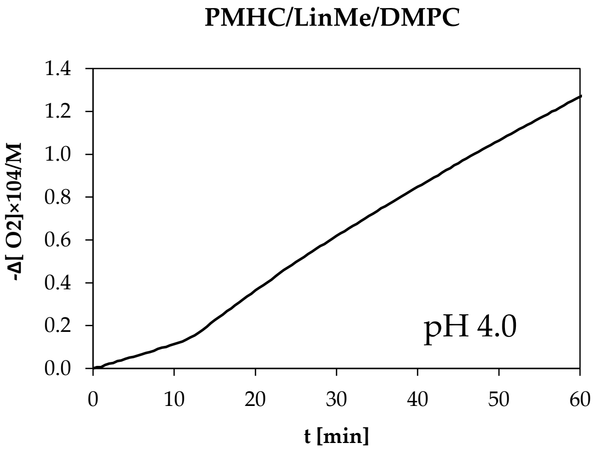

Figure S16. Typical plot of the oxygen uptake for ABAP-initiated autoxidation of LinMe (2.74 mM) in DMPC (20.2 mM) liposome in presence of PMHC (1 μM). Measurement were carried out at 37°C at pH 4.0.

Table S2. The lengths of induction periods, τ_{ind}, the rates of initiation, R_i, kinetic chain length, v_{ox}, v_{inh}, v_{ox1} and the inhibition rate constants, k_{inh}, determined for peroxidation of MeLin/DMPC liposome inhibited by 1 µM PMHC. Experiments were performed in 20,2 mM DMPC liposome with 2.74 mM MeLin at 37°C at pH 4.0, Peroxidation was initiated by 10 mM ABAP. All experiments were run at 3-6 times. Values were expressed as the mean ± standard deviation (SD).

pH	τ /min	R_i /nMs^{-1}	R_{inh} /nM^{-1}	$k_{inh} \times 10^{-3}$ /M^{-1}s^{-1}	$R_{ox} \times 10^7$ /M^{-1}	$R_{ox1} \times 10^7$ /M^{-1}	v_{ox}[a]	v_{inh}[a]	v_{ox1}[a]
4.0	10.9±0.6	3.1	18.1±2.8	12.8±3.6	0.8±0.1	0.4±0.1	26	6	14

[a] The kinetic chain length v is the number of peroxidation cycles triggered by one initiating radical. Here, for non-inhibited peroxidation, $v_{ox1}=R_{ox1}/R_i$
